# SAR Analyzer: a tool for interactive SAR data visualization and navigation

**DOI:** 10.1186/1758-2946-4-S1-P31

**Published:** 2012-05-01

**Authors:** Lisa Peltason, Daniel Stoffler

**Affiliations:** 1 Therapeutic Modalities Informatics / Cheminformatics and Statistics, Pharma Research & Early Development Informatics, F. Hoffmann-La Roche Ltd, CH-4070 Basel, Switzerland

## 

The analysis of structure-activity relationships (SAR) is a central task in medicinal chemistry. The association between chemical structures of biologically active molecules and multiple property and assay data provides the basis for selection, optimization, and evaluation of potential drug candidate molecules.

In an endeavour to facilitate the navigation of complex data landscapes and enable intuitive access to SAR information, an interactive SAR analysis platform is being developed at Roche. Focusing on information-rich data visualizations, we aim at supporting the medicinal chemists in their decision-making process, rather than making quantitative or qualitative predictions. The “SAR Analyzer” integrates cutting-edge visualization techniques and enhances them with key capabilities such as real-time interaction with the data, an intuitive user interface, and functionality to easily extract and navigate multi-property data.

Here we show how two complementary approaches are integrated in the SAR Analyzer. The “SAR Map” visualization provides a holistic view of the distribution of molecular structures and properties in a data set [1]. Molecules are displayed in a 2D map projection based on chemical features or similarity. Color shading indicates the distribution of selected molecular properties or biological activity. By contrast, the “SAR Tree” visualization [2] focuses on subsets of similar compounds. Starting at a user-selected reference compound, all molecules within a similarity radius are organized in a hierarchical tree structure that makes it possible to interactively browse different compound series and derive and test SAR hypotheses.

Both concepts depart from classical SAR data analysis and support scientific reasoning by making SAR information accessible in an intuitive visual way. Linking both approaches allows the analysis of SAR data on different levels of detail and helps to address questions that are relevant in different stages of a medicinal chemistry research project.
